# Systemic Inflammation in Metabolic Syndrome: Increased Platelet and Leukocyte Activation, and Key Role of CX_3_CL1/CX_3_CR1 and CCL2/CCR2 Axes in Arterial Platelet-Proinflammatory Monocyte Adhesion

**DOI:** 10.3390/jcm8050708

**Published:** 2019-05-18

**Authors:** Patrice Marques, Aida Collado, Sergio Martinez-Hervás, Elena Domingo, Esther Benito, Laura Piqueras, José T. Real, Juan F. Ascaso, Maria-Jesus Sanz

**Affiliations:** 1 Department of Pharmacology, University of Valencia, Av. Blasco Ibáñez 15, 46010 Valencia, Spain; patricegmarques@gmail.com (P.M.); aida.collado@uv.es (A.C.); eledo2@hotmail.com (E.D.); laura.piqueras@uv.es (L.P.); 2 Institute of Health Research INCLIVA, Av. Menéndez Pelayo 4, 46010 Valencia, Spain; Sergio.Martinez@uv.es (S.M.-H.); esther.benito@hotmail.com (E.B.); Jose.T.Real@uv.es (J.T.R.); 3 Department of Medicine, Faculty of Medicine and Odontology, University of Valencia, Av. Blasco Ibáñez 15, 46010 Valencia, Spain; 4 Endocrinology and Nutrition Service, University Clinic Hospital of Valencia, Av. Menéndez Pelayo 4, 46010 Valencia, Spain; 5 CIBERDEM-Spanish Biomedical Research Centre in Diabetes and Associated Metabolic Disorders, ISCIII, Av. Monforte de Lemos 3-5, 28029 Madrid, Spain

**Keywords:** metabolic syndrome, cytokines, chemokines, leukocyte activation, platelet activation, endothelial dysfunction, systemic inflammation

## Abstract

Background: Metabolic syndrome is associated with low-grade systemic inflammation, which is a key driver of premature atherosclerosis. We characterized immune cell behavior in metabolic syndrome, its consequences, and the potential involvement of the CX_3_CL1/CX_3_CR1 and CCL2/CCR2 chemokine axes. Methods: Whole blood from 18 patients with metabolic syndrome and 21 age-matched controls was analyzed by flow cytometry to determine the leukocyte immunophenotypes, activation, platelet-leukocyte aggregates, and CX_3_CR1 expression. ELISA determined the plasma marker levels. Platelet-leukocyte aggregates adhesion to tumor necrosis factor-α (TNFα)-stimulated arterial endothelium and the role of CX_3_CL1/CX_3_CR1 and CCL2/CCR2 axes was investigated with the parallel-plate flow chamber. Results: When compared with the controls, the metabolic syndrome patients presented greater percentages of eosinophils, CD3^+^ T lymphocytes, Mon2/Mon3 monocytes, platelet-eosinophil and -lymphocyte aggregates, activated platelets, neutrophils, eosinophils, monocytes, and CD8^+^ T cells, but lower percentages of Mon1 monocytes. Patients had increased circulating interleukin-8 (IL-8) and TNFα levels and decreased IL-4. CX_3_CR1 up-regulation in platelet-Mon1 monocyte aggregates in metabolic syndrome patients led to increased CX_3_CR1/CCR2-dependent platelet-Mon1 monocyte adhesion to dysfunctional arterial endothelium. Conclusion: We provide evidence of generalized immune activation in metabolic syndrome. Additionally, CX_3_CL1/CX_3_CR1 or CCL2/CCR2 axes are potential candidates for therapeutic intervention in cardiovascular disorders in metabolic syndrome patients, as their blockade impairs the augmented arterial platelet-Mon1 monocyte aggregate adhesiveness, which is a key event in atherogenesis.

## 1. Introduction

Cardiovascular disease (CVD), especially coronary heart disease and stroke, are the main causes of mortality in most European countries [1], and atherosclerosis is the principal contributor to the pathogenesis of myocardial and cerebral ischemic disorders. It has become increasingly evident that systemic inflammation is the main driver of premature atherosclerosis and its complications. Against this background, there is evidence supporting that low-grade systemic inflammation is often associated with the metabolic syndrome [2], which is a cluster of cardiovascular risk factors with a high prevalence in Western countries [3]. The diagnosis of metabolic syndrome is made when at least three of the five following criteria are met: (1) abdominal obesity; (2) arterial hypertension; (3) dysglycemia; (4) hypertriglyceridemia; and, (5) low high-density lipoprotein (HDL) cholesterol levels [4]. These pathophysiological changes can lead to oxidative stress and inflammation, which increases the risk for atherosclerosis and CVD [5,6,7,8,9,10]. Indeed, clinical evidence suggests that metabolic syndrome promotes endothelial dysfunction [5], a prothrombotic and proinflammatory state that precedes atherogenic events [11].

Several studies have shown that soluble inflammatory markers, including tumor necrosis factor-α (TNFα), interleukin-6 (IL-6), monocyte chemoattractant protein-1 (MCP-1/CCL2), CXCL16, and high-sensitivity C-reactive protein are found at higher levels in patients with metabolic syndrome than in the age-matched controls [12,13,14,15]. Moreover, metabolic syndrome has been related to an increased number of several circulating leukocyte subsets and enhanced monocyte and platelet activation [12,16,17,18]. Nonetheless, some results on the inflammatory status of this complex metabolic disorder are contentious, particularly with regard to the soluble mediators and leukocyte subsets [13,17,18,19,20]. Therefore, deeper knowledge regarding the different cellular and soluble components of systemic inflammatory responses that are associated with metabolic syndrome and their clinical consequences is needed.

The CX_3_CL1/CX_3_CR1 and CCL2/CCR2 chemokine axes have been implicated in the development of CVD [21,22,23,24]. Fractalkine/CX_3_CL1 is a transmembrane protein that is widely expressed in immune and non-immune human cells that occurs in two distinct for metabolic syndrome: a membrane-bound form that promotes the firm adhesion of cells expressing its cognate receptor CX_3_CR1, and a soluble form that is generated by the proteolytic cleavage of the transmembrane form [25]. The expression of CX_3_CL1 is markedly upregulated in human arterial and venous endothelial cells during the inflammatory process [25,26]. It is also known that the CX_3_CL1/CX_3_CR1 axis is involved in mononuclear cell attachment to endothelium and the subsequent transmigration of monocytes and lymphocytes, a critical step in the atherogenic process [25,26].

MCP-1/CCL2 is a chemokine that is produced by several cell types, including endothelial cells, fibroblasts, epithelial cells, smooth muscle cells, and monocytes/macrophages [27], and it regulates the migration and infiltration of monocytes, T lymphocytes, and NK cells through interaction with its receptor (CCR2). Accordingly, MCP-1/CCL2 is considered as a potential intervention target in atherosclerosis and diabetes with insulin resistance [27,28,29].

We performed a comprehensive analysis of different cellular and soluble immune players in patients with metabolic syndrome and examined their potential consequences for arterial leukocyte adhesion, given that a better understanding of immune system behavior might open new horizons for CVD prognosis and treatment. As increased circulating levels of TNFα have been detected in metabolic syndrome patients [12,15], which can up-regulate CX_3_CL1 expression and promote the generation and release of CCL2 [30,31], we also investigated the role of CX_3_CL1/CX_3_CR1 and CCL2/CCR2 axes in platelet-leukocyte and leukocyte adhesion to the dysfunctional arterial endothelium in a metabolic syndrome model. 

## 2. Materials and Methods

### 2.1. Cell Culture

The human umbilical arterial endothelial cells (HUAEC) were isolated by collagenase treatment. Details are described in the Supplemental data.

### 2.2. Human Study Populations

A total of 39 subjects (18 patients with metabolic syndrome and 21 age-matched control subjects) were included in the present study. The Endocrinology Unit at the University Clinic Hospital of Valencia recruited the patients and controls (Valencia, Spain). All of the procedures involving human samples complied with the principles outlined in the Declaration of Helsinki and the institutional ethics committee of the University Clinic Hospital, Valencia approved them. All of the subjects signed an informed consent. 

The general inclusion criteria for entering in the study were: male and female, age 18–65 years.

Inclusion criteria for subjects with metabolic syndrome: medical history, clinical exam, and biochemical parameters, according to the definition established by the International Diabetes Federation confirmed metabolic syndrome. The diagnostic criteria for metabolic syndrome were defined by at least three of the following conditions: (1) Male waist circumference ≥ 94 cm, female waist circumference ≥ 80 cm; (2) triglycerides (TG) ≥ 150 mg/dL or pharmacological treatment for hypertriglyceridemia; (3) Blood pressure ≥ 130 (systolic) and/or 85 (diastolic) mmHg or pharmacological treatment for hypertension; (4) Fasting glucose level ≥ 100 mg/dL; and, (5) Male HDL-cholesterol < 40 mg/dL, female HDL-cholesterol < 50 mg/dL. 

Inclusion criteria for controls: Healthy volunteers were recruited among personal and plasma donors of our center. All of them were normolipidemic (fasting total cholesterol and triglycerides below the 90th percentile of our population), normoglycemic (fasting glucose < 100 mg/dL), and non-obese (BMI < 30 kg/m^2^).

Exclusion criteria: BMI > 35 kg/m^2^, type 2 diabetes, personal history of major vascular events (coronary artery disease, peripheral arterial disease, or stroke), abnormal intima/media thickness, hypothyroidism, asthma, autoimmune diseases, chronic hepatic disease, chronic renal failure, chronic heart failure (NYHA > II), cancer, pregnancy or breast-feeding, infection or inflammatory disease (including personal history of allergy) in the six weeks prior to the study, the use of drugs that are capable of modifying the lipid profile or inflammation that cannot be withdrawn six weeks before starting the study (lipid-lowering drugs, glucose-lowering drugs, anti-hypertensive drugs, anti-inflammatory drugs and antioxidant supplements), and alcohol consumption > 30 g per day. 

Table 1 shows the clinical and biological parameters of patients and age-matched controls.

### 2.3. Flow Cytometry

Full details are described in the Supplemental data. Figures S1–S5 and Table S1 describe the gating strategies.

### 2.4. Measurement of Soluble Metabolic and Inflammatory Markers

Heparinized whole blood was collected from patients and age-matched controls, and soluble metabolic and inflammatory markers were measured by ELISA in plasma. Further details are described in the Supplemental data.

### 2.5. Leukocyte-Endothelial Cell Interactions under Flow Conditions

Whole blood treated or not with ethylenediaminetetraacetic acid (EDTA), was perfused across endothelial monolayers that were unstimulated or stimulated with TNFα (20 ng/mL), for 24 h. In some experiments, the cells were pre-incubated with a neutralizing antibody against CX_3_CL1 or CCL2 or with an isotype-matched control antibody. Details are described in the Supplemental data.

### 2.6. Statistical Analysis

All of the results were analyzed using GraphPad Prism software (GraphPad Software, Inc., La Jolla, CA, USA). The values are expressed as individual data points, percentages, or mean ± standard error of the mean (SEM) when appropriate. For two-group comparisons, paired or unpaired Student’s t test was used in data that passed both normality (Kolmogorov-Smirnov) and equal variance (Levene) tests, as appropriate; otherwise, the non-parametric Mann–Whitney U test was performed. For comparisons among multiple groups, one-way analysis of variance, followed by *post hoc* Bonferroni analysis, was used in data that passed both normality and equal variance tests; otherwise, the non-parametric Kruskal–Wallis test followed by Dunn’s *post hoc* analysis was used. Data was considered to be statistically significant at *p* < 0.05.

## 3. Results

A total of 39 subjects (18 patients with metabolic syndrome and 21 age-matched controls) were included in the present study. Table 1 shows the demographic, clinical, and laboratory characteristics of patients and controls. No statistically significant differences were found regarding the age, gender, or height between the two groups. By contrast, body mass index (BMI), weight, waist circumference, systolic and diastolic blood pressure, glucose, insulin levels, homeostatic model assessment (HOMA) index, levels of total cholesterol, low-density lipoprotein (LDL)-cholesterol, and triglycerides were all significantly higher in metabolic syndrome patients than in the controls, whereas high-density lipoprotein (HDL)-cholesterol levels were significantly lower, as expected. Indeed, patients with metabolic syndrome had insulin resistance and abdominal obesity. Additionally, a table indicating the number of patients that met each of the five metabolic syndrome criteria is shown in the Supplemental Data (Table S2). In addition, none of the sub-groups markedly differ from the healthy group in age or other non-metabolic syndrome parameters (Table S2, Supplemental Data).

### 3.1. Platelet Activation is Enhanced in Patients with Metabolic Syndrome

**Figure 1:**
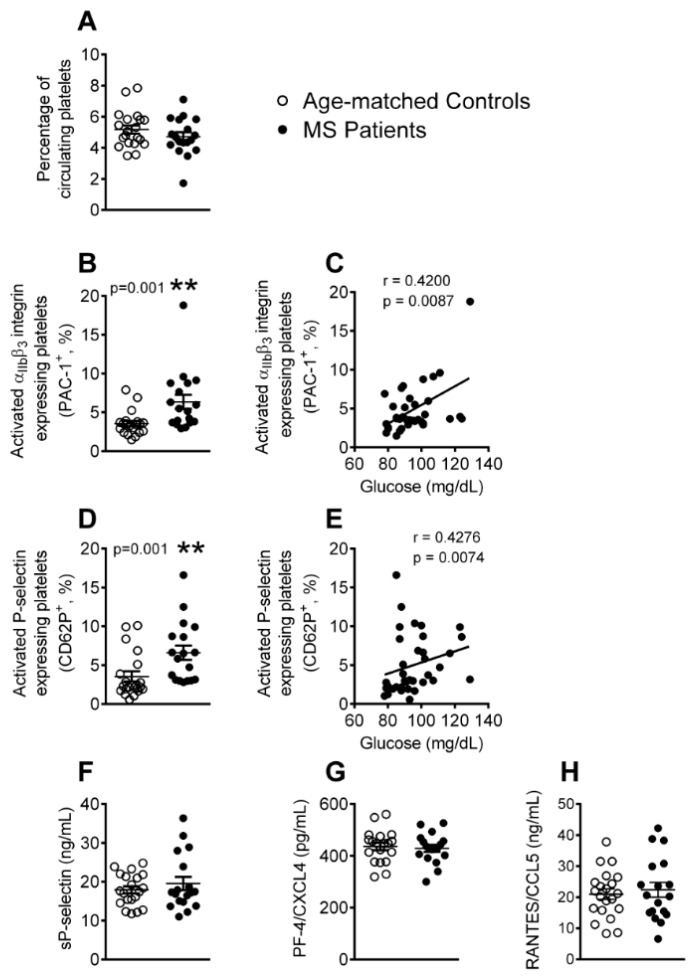
Platelet activation is elevated in patients with metabolic syndrome. Flow cytometry analysis of platelets stained with conjugated antibodies against CD41 (**A**), CD41 and PAC-1 (**B**), and CD41 and P-selectin (**D**). Results are expressed as percentage of positive cells. Soluble (s)P-selectin (**F**), platelet factor-4 (PF-4/CXCL4) (**G**) and regulated on activation normal T cell expressed and secreted (RANTES/CCL5) (**H**) plasma levels (ng or pg/mL) were measured by enzyme-linked immunosorbant assay (ELISA). (*n* = 21 control subjects and *n* = 18 metabolic syndrome patients). Values are expressed as mean ± SEM. ** *p* < 0.01 relative to values in the control group. Correlations between circulating glucose levels and percentage of circulating platelets PAC-1^+^ (**C**) or P-selectin^+^ (**E**).

As a first step, we determined the platelet activation state and the levels of platelet activation-related soluble mediators in the blood samples from the two groups using flow cytometry and enzyme-linked immunosorbant assay (ELISA). While no significant differences in the number of circulating platelets were found between the two groups (Figure 1A), the percentage of platelets expressing PAC-1 and P-selectin (CD62P) was significantly higher in the metabolic syndrome patients than in the controls (Figure 1B,D), indicating an enhanced activation state. In addition, the increased percentage of activated platelets positively correlated with the blood glucose levels (Figure 1C,E). P-selectin can translocate to the cell surface upon cell activation, where it can be cleaved and released into the circulation as soluble P-selectin (sP-selectin). However, we found no differences in the circulating levels of sP-selectin between the two groups (Figure 1F). Likewise, circulating levels of platelet chemokines that can be released upon their activation, such as platelet factor-4 (PF-4/CXCL4) and regulated on activation normal T cell expressed and secreted (RANTES/CCL5), were similar in both of the groups (Figure 1G,H).

### 3.2. The Percentage of Activated Neutrophils and Circulating Levels of IL-8 Are Elevated in Patients with Metabolic Syndrome

**Figure 2:**
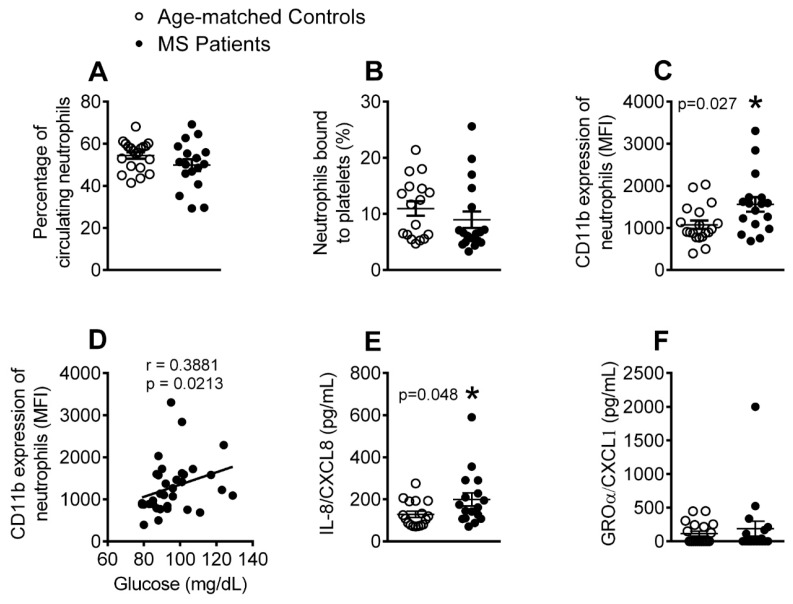
The percentage of activated neutrophils and IL-8 circulating levels are increased in patients with metabolic syndrome. Flow cytometry analysis of heparinized whole blood co-stained with specific markers for platelets and neutrophils (**A**,**B**). Neutrophils were also stained for CD11b (**C**). Results are expressed as percentage of positive cells and as mean of fluorescence intensity (MFI). Interleukin-8 (IL-8)/CXCL8 (**E**) and growth-regulated oncogene-α (GROα/CXCL1) (**F**) plasma levels (pg/mL) were measured by ELISA (*n* = 21 age-matched controls and *n* = 18 metabolic syndrome patients). Values are expressed as mean ± SEM. * *p* < 0.05 relative to values in the control group. Correlations between circulating glucose levels and CD11b expression on neutrophils (**D**).

We next evaluated several parameters related to the activation of different leukocyte subsets. No differences were detected between the groups in the number of circulating neutrophils (Figure 2A) or in the percentage of neutrophil-platelet aggregates (Figure 2B). However, neutrophil activation (CD11b expression) was higher in metabolic syndrome patients than in the age-matched controls (Figure 2C), and was positively correlated with circulating glucose levels (Figure 2D). Given that chemokines, such as growth-regulated oncogene-α (GROα/CXCL1) and IL-8 (CXCL8) can induce human neutrophil activation and chemotaxis, we quantified their levels in plasma. Although, metabolic syndrome patients presented higher plasma levels of IL-8 than the age-matched controls, no differences were found in the circulating levels of GROα/CXCL1 (Figure 2E,F).

### 3.3. Patients with Metabolic Syndrome Present a Higher Percentage of Circulating Eosinophils, Increased Number of Eosinophil-Platelet Aggregates, Enhanced Eosinophil Activation and Decreased Eotaxin-2/CCL24 Plasma Levels

**Figure 3:**
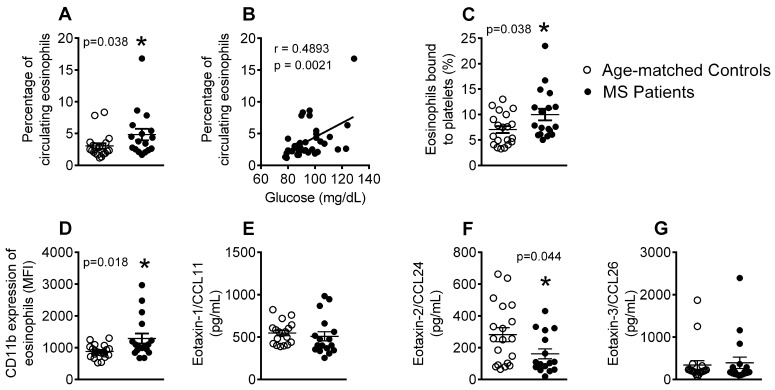
The percentage of circulating eosinophils, platelet-eosinophil aggregates, and activated eosinophils is increased in patients with metabolic syndrome. Flow cytometry analysis of heparinized whole blood co-stained with specific markers for platelets and eosinophils (**A**,**C**). The eosinophils were also stained for CD11b (**D**). Results are expressed as percentage of positive cells and as mean of fluorescence intensity (MFI). Eotaxin-1/CCL11 (**E**), eotaxin-2/CCL24 (**F**), and eotaxin-3/CCL26 (**G**) plasma levels (pg/mL) were measured by ELISA (*n* = 21 age-matched controls and *n* = 18 metabolic syndrome patients). Values are expressed as mean ± SEM. * *p* < 0.05 relative to values in the control group. Correlations between circulating glucose levels and percentage of circulating eosinophils (**B**).

An increase in the number of circulating eosinophils has been previously reported in patients with metabolic syndrome [10]. In agreement with this observation, we found that the patients with metabolic syndrome presented a higher percentage of circulating eosinophils relative to the age-matched controls, and this positively correlated with blood glucose levels (Figure 3A,B). Additionally, an increase in the percentage of eosinophil-platelet aggregates (Figure 3C), as well as enhanced eosinophil activation (CD11b expression, Figure 3D), was found in the metabolic syndrome group. As different chemokines are involved in eosinophil activation, with eotaxin-1/CCL11, -2/CCL24, and -3/CCL26 being especially relevant [32], we also examined their circulating levels. Whereas, no differences in the plasma levels of eotaxin-1/CCL11 and eotaxin-3/CCL26 were detected between the two groups (Figure 3E,G), circulating levels of eotaxin-2/CCL24 were significantly lower in the metabolic syndrome group than in the controls (Figure 3F).

### 3.4. Patients with Metabolic Syndrome Present a Higher Percentage of Circulating Monocytes and Enhanced Monocyte Activation

**Figure 4:**
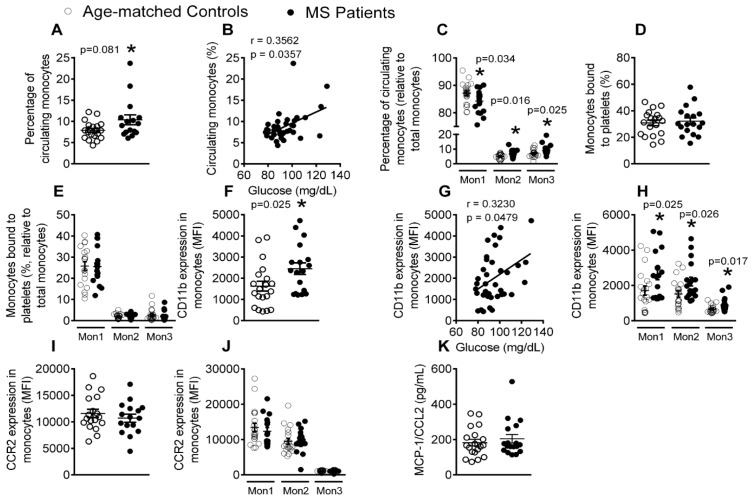
The percentage of total circulating monocytes, Mon2 and Mon 3 monocytes and activated Mon1–3 monocytes is elevated in patients with metabolic syndrome, whereas the percentage of Mon1 monocytes is decreased. Flow cytometry analysis of heparinized whole blood co-stained with specific markers for platelets and Mon1, 2, and 3 monocytes (**A**,**C**–**E**), CD11b integrin (**F**,**H**) and CCR2 (**I**,**J**). Results are expressed as percentage of positive cells or mean fluorescence intensity (MFI). MCP-1/CCL2 (**K**) plasma levels (pg/mL) were measured by ELISA (*n* = 21 control subjects and *n* = 18 metabolic syndrome patients). Values are expressed as mean ± SEM. * *p* < 0.05 relative to values in the control group. Correlations between circulating glucose levels and percentage of circulating monocytes (**B**) as well as CD11b expression on monocytes (**G**).

Next, we analyzed monocyte subpopulations in peripheral blood by flow cytometry, finding that the percentage of total circulating monocytes was higher in metabolic syndrome patients than in the control subjects, which, again, positively correlated with the levels of circulating glucose (Figure 4A,B). Monocytes can be divided into three subsets with distinct features, including their differential expression of the cell surface markers CD14, CD16, and CCR2 (Table S1, Supplemental data). We examined for changes in these subsets in the two groups, finding that, whereas the percentage of circulating type 1 monocytes (Mon1) was significantly lower in metabolic syndrome patients than in controls, the percentage of type 2 (Mon2) and type 3 (Mon3) monocytes was significantly higher (Figure 4C). No differences in the percentage of monocyte-platelet aggregates were found between the groups (Figure 4D,E), in accord with the results for neutrophil-platelet aggregates. When monocyte activation (CD11b expression) was evaluated, all of the monocyte subsets were found to be in an activated state in metabolic syndrome patients (Figure 4F,H), which positively correlated with glucose levels (Figure 4G). However, neither CCR2 expression on monocytes nor the plasma levels of its cognate ligand MCP-1/CCL2 differed between the groups (Figure 4I–K).

### 3.5. The Percentages of Circulating Lymphocytes, Lymphocyte-Platelet Aggregates and Activated CD8^+^ Lymphocytes Are Elevated in Patients with Metabolic Syndrome

**Figure 5:**
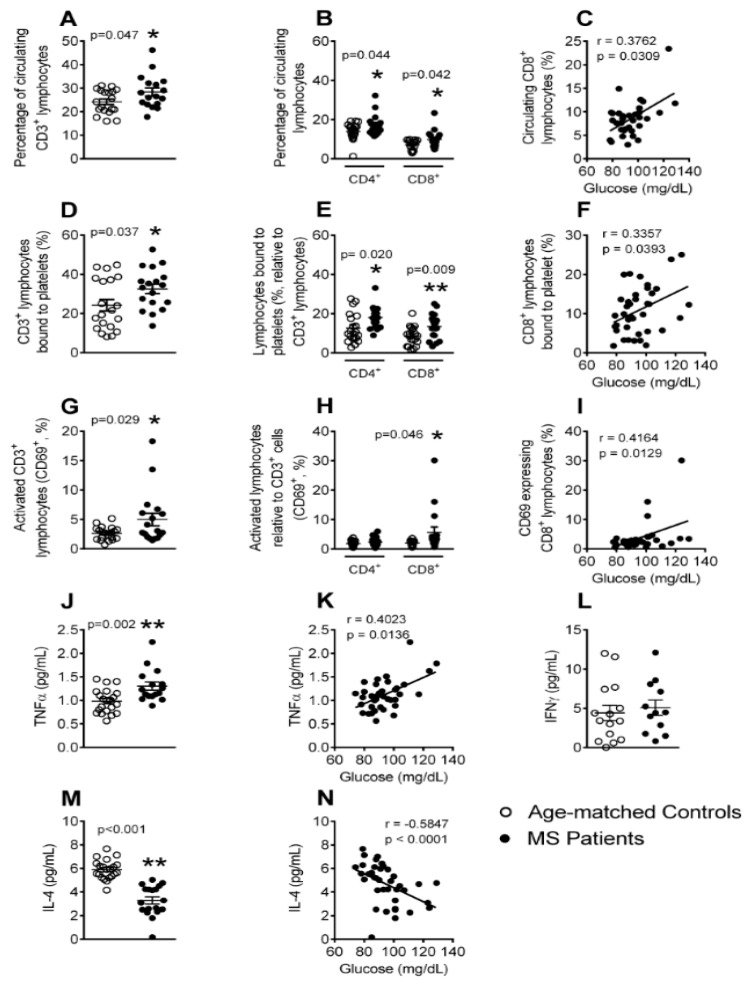
The percentage of circulating CD3^+^ lymphocytes (CD4^+^ and CD8^+^), platelet-CD3^+^ lymphocyte (CD4^+^ and CD8^+^) aggregates, CD8^+^ lymphocyte activation, and tumor necrosis factor-α (TNFα) circulating levels, are significantly elevated in patients with metabolic syndrome, whereas the plasma levels of IL-4 are decreased. Heparinized whole blood was co-stained with specific markers for platelets and CD3^+^, CD4^+^, and CD8^+^ lymphocytes (**A**,**B**,**D**,**E**), as well as for CD69 (**G**,**H**). Results are expressed as the percentage of positive cells. TNFα (**J**), IFNγ (**L**) and IL-4 (**M**) plasma levels (pg/mL) were measured by ELISA (*n* = 21 control subjects and *n* = 18 metabolic syndrome patients). Values are expressed as mean ± SEM. * *p* < 0.05 or ** *p* < 0.01 relative to values in the control group. Correlations between circulating glucose levels and the percentage of circulating CD8^+^ cells (**C**), platelet-CD8^+^ lymphocyte aggregates (**F**), activated CD8^+^ lymphocytes (**I**), as well as TNFα (**K**) and IL-4 (**N**) circulating levels.

Mature T cells express the general co-receptor CD3, and also express either CD4 (T helper cell) or CD8 (cytotoxic T cell) epitopes, depending on the type of T cell. Flow cytometry analysis showed that metabolic syndrome patients presented higher percentages of circulating CD3^+^, CD4^+^, and CD8^+^ lymphocytes than controls (Figure 5A,B), and the percentage of CD8^+^ lymphocytes positively correlated with circulating glucose levels (Figure 5C). Moreover, the percentage of CD3^+^, CD4^+^, and CD8^+^ lymphocytes bound to platelets (Figure 5D,E), and also the activation state (CD69 expression) of CD3^+^ and CD8^+^ lymphocytes (Figure 5G,H) was greater in patients than in control subjects. Interestingly, a positive correlation was found between the percentage of CD8^+^ lymphocyte-platelet aggregates and CD8^+^CD69^+^ cells with plasma glucose levels (Figure 5F,I). TNFα is a central adipokine in metabolic syndrome [12,15], and we found an increase in its circulating levels in metabolic syndrome patients (Figure 5J), which again positively correlated with the circulating glucose concentrations (Figure 5K). By contrast, whereas the circulating levels of IFNγ, a cytokine that is released by Th1 lymphocytes, were not different between patients and controls (Figure 5L), the anti-inflammatory cytokine IL-4, which Th2 lymphocytes mainly produce, was significantly lower in the circulation of metabolic syndrome patients (Figure 5M). Indeed, an inverse correlation was found between IL-4 and circulating glucose levels (Figure 5N). The plasma levels of the lymphocyte-associated cytokines IL-6, IL-10, IL-12, IL-13, IL-25, and IL-33 were also measured, but no differences were found between the groups (data not shown).

### 3.6. Enhanced CX_3_CR1 Expression in Platelets, Different Leukocyte Subsets and Leukocyte-Platelet Aggregates in Patients with Metabolic Syndrome

**Figure 6:**
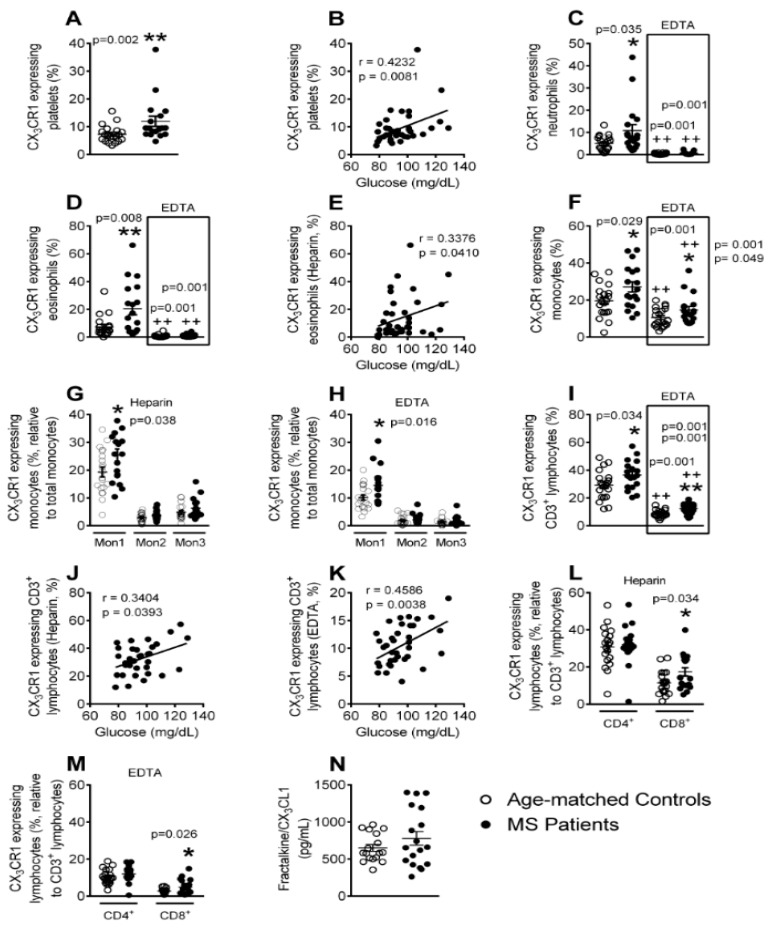
Enhanced CX_3_CR1 expression on platelets, platelet-neutrophil, -eosinophil, -Mon1, -CD3^+^CD8^+^ lymphocyte aggregates, Mon1 monocytes and CD3^+^CD8^+^ lymphocytes in metabolic syndrome patients. Flow cytometry analysis of heparinized and (ethylenediaminetetraacetic acid) EDTA-treated whole blood co-stained with specific markers for CX_3_CR1^+^ and platelets, neutrophils, eosinophils, Mon1, 2 and 3 monocytes as well as CD3^+^, CD4^+^ and CD8^+^ lymphocytes (**A**–**D**,**F**–**I**,**L**,**M**). Results are expressed as percentage of positive cells. Fractalkine/CX_3_CL1 (**N**) plasma levels (pg/mL) were measured by ELISA (*n* = 21 control subjects and *n* = 18 metabolic syndrome patients). Values are expressed as mean ± SEM. * *p* < 0.05 or ** *p* < 0.01 relative to values in the control group; ++*p* < 0.01 relative to values in the respective heparin group. Correlations between circulating glucose levels and CX_3_CR1-expressing platelets (**B**), CX_3_CR1-expressing platelet-eosinophil aggregates (**E**), CX_3_CR1-expressing platelet-CD3^+^ lymphocyte aggregates (**J**), and CX_3_CR1-expressing CD3^+^ lymphocytes (**K**).

The CX_3_CL1/CX_3_CR1 axis is involved in leukocyte recruitment and in the development of cardiovascular disorders [24,25,26,33]. Thus, we next evaluated CX_3_CR1 expression in platelets, leukocyte subtypes, and leukocyte-platelet aggregates of both groups by flow cytometry. We found that the percentage of CX_3_CR1-expressing platelets was significantly higher in the metabolic syndrome group than in the controls and positively correlated with blood glucose levels (Figure 6A,B). Although both neutrophil- and eosinophil-platelet aggregates of metabolic syndrome patients showed increased CX_3_CR1 expression when compared with the control group (Figure 6C,D, Heparin), neither expressed CX_3_CR1 after platelet dissociation (Figure 6C,D, EDTA). The percentage of the CX_3_CR1^+^ eosinophil-platelet aggregates positively correlated with circulating glucose levels (Figure 6E). Additionally, in the total monocyte subpopulation, an increase in the percentage of CX_3_CR1-expressing cells was evident in the metabolic syndrome patients, irrespective of whether platelets were bound or unbound (Figure 6F). Detailed analysis of this population revealed that the Mon1 monocytes were responsible for these differences (Figure 6G,H). A similar profile was detected in CD3^+^ lymphocytes from metabolic syndrome patients (Figure 6I) and the percentage of CD3^+^CX_3_CR1^+^ lymphocytes was found to positively correlate with blood glucose levels, with or without bound platelets (Figure 6J,K). This enhanced CX_3_CR1 expression in CD3^+^ lymphocytes was due to CD8^+^, and not CD4^+^ lymphocytes (Figure 6L,M). In spite of these findings, the circulating levels of soluble fractalkine/CX_3_CL1 ligand did not differ between the two groups (Figure 6N). 

### 3.7. Circulating Leukocytes from Patients with Metabolic Syndrome Show Superior Adhesiveness to TNFα-Stimulated Arterial Endothelium, Which is Partly Dependent on CX_3_CL1 and CCL2 Activity

**Figure 7:**
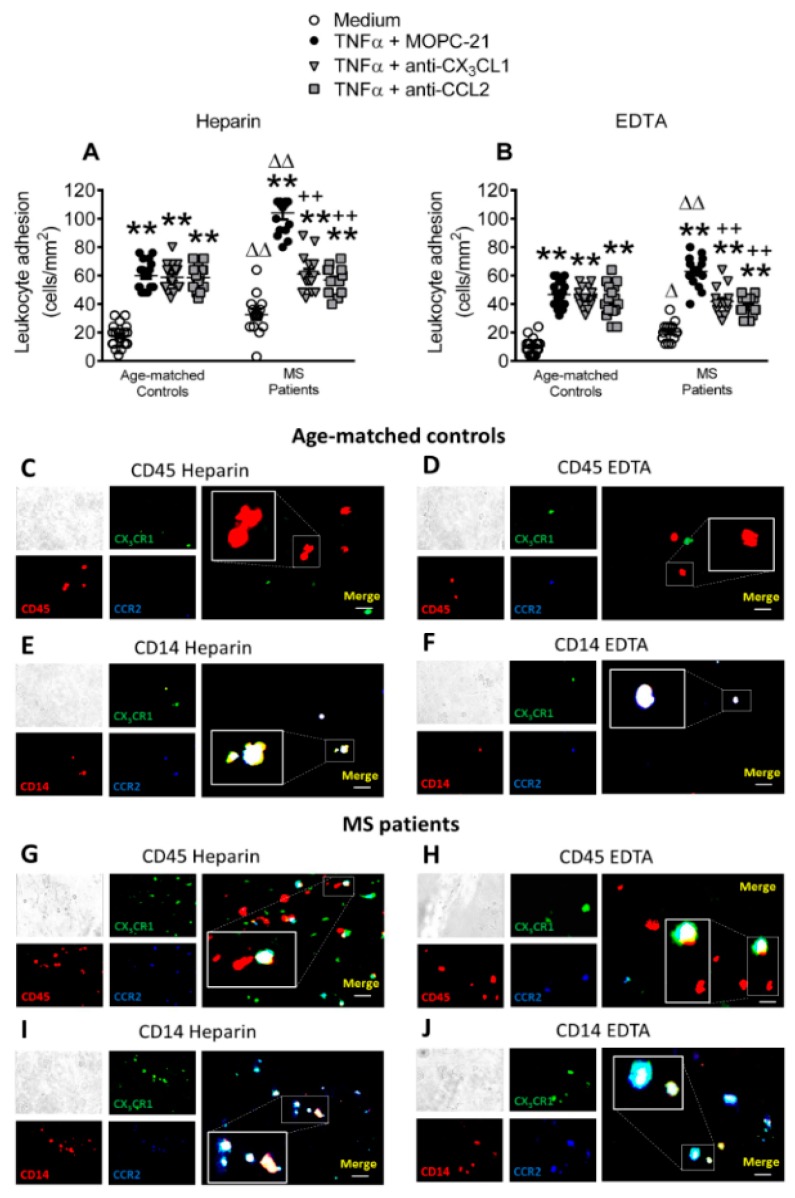
Circulating leukocytes from metabolic syndrome patients show increased adhesiveness to TNFα-stimulated HUAECs, which is partly dependent on both CX_3_CL1/CX_3_CR1 and CCL2/CCR2 interactions. HUAEC were stimulated with TNFα (20 ng/mL) for 24 h. Some of the cells were incubated with a CX_3_CL1 or a CCL2 neutralizing antibody or an isotype-matched control antibody (MOPC-21). Subsequently, whole blood from metabolic syndrome patients and age-matched controls incubated without (**A**) or with (**B**) EDTA was perfused across endothelial monolayers for 7 min at 0.5 dyn/cm^2^ and leukocyte adhesion quantified (*n* = 21 control subjects and *n* = 18 metabolic syndrome patients). Results are presented as leukocyte adhered per mm^2^ (cells/mm^2^). Values are expressed as mean ± SEM. ** *p* < 0.01 relative to values in medium; ++*p* < 0.01 relative to TNFα+MOPC-21; ∆*p* < 0.05 or ∆∆*p* < 0.01 relative to the respective age-matched control group. Immunofluorescence analysis showing adherent platelet–leukocyte cell complexes, platelet-monocyte aggregates, leukocytes or monocytes to TNFα-stimulated HUAEC (**C**–**J**). Heparinized blood from age-matched controls and patients with metabolic syndrome was incubated without or with EDTA. After the flow chamber assay, cells were fixed with 4% paraformaldehyde and blocked in PBS containing 1% BSA. Subsequently, cells were incubated for 2 h with an Alexa 594-conjugated antibody against human CD45 to detect leukocytes (1:50 dilution, red, **C**,**D**,**G**,**H**) or an APC-conjugated antibody against human CD14, to detect monocytes (1:50 dilution, red, **E**,**F**,**I**,**J**). Additionally, all of the cells (**C**–**J**) were incubated for 2 h with an Alexa Fluor 350-conjugated antibody against human CCR2 (1:50 dilution, blue) and a FITC-conjugated antibody against human CX_3_CR1 (1:50 dilution, green). Images were captured with a Zeiss Axio Observer A1 fluorescence microscope. Bar graph = 50 μm.

We tested the role of the CX_3_CL1/CX_3_CR1 and CCL2/CCR2 axes on leukocyte adhesion to dysfunctional arterial endothelium (HUAEC monolayers) under dynamic flow conditions to explore the functional consequences of these observations. We used TNFα as an inflammatory stimulus to mimic the dysfunctional endothelium, as it is a key cytokine in metabolic syndrome [12,15], and its circulating levels are elevated in metabolic syndrome patients (Figure 5J). Additionally, TNFα induces endothelial CX_3_CL1 expression [25] and CCL2 generation [34]. Leukocyte adhesiveness was greater in the metabolic syndrome group than in the control group when samples of heparinized whole blood from metabolic syndrome patients and age-matched controls were perfused across TNFα-stimulated HUAEC (Figure 7A). Notably, the neutralization of CX_3_CL1 or CCL2 activity on HUAEC led to a decrease in platelet-leukocyte endothelial adhesion in the metabolic syndrome group only after TNFα stimulation of HUAEC (Figure 7A). 

When the platelets were disaggregated from leukocytes with EDTA, leukocyte adhesion to endothelial cells remained significantly higher in the metabolic syndrome group than in the control group (Figure 7B), despite the significantly lower number of leukocytes that adhered to TNFα-stimulated HUAEC than when platelets were bound (Figure 7A, Heparin). The neutralization of either CX_3_CL1 or CCL2 activity on HUAEC again markedly reduced TNFα-induced leukocyte adhesion in the metabolic syndrome group, but not in the control group (Figure 7B). Consistent with these observations, immunofluorescence studies revealed enhanced adherent platelet-leukocyte complexes to TNFα-stimulated endothelial cells from metabolic syndrome patients when compared with age-matched controls and some of these adhered complexes expressed both CCR2 and CX_3_CR1 receptors (Figure 7C,E,G,I). Further analysis of the monocyte subpopulation (CD14^+^) showed that most of them expressed both chemokine receptors (Figure 7E,I). Of note, when the platelets were disaggregated from leukocytes with EDTA, leukocyte adhesion to TNFα-stimulated HUAEC was evidently diminished, but this parameter was markedly greater in the metabolic syndrome group than the control group and again monocytes mainly expressed CCR2 and CX_3_CR1 receptors (Figure 7D,F,H,J).

## 4. Discussion

Metabolic syndrome is associated with an increased risk of developing arteriosclerosis and serious ischemic events [5,6,7,8,9,10]. Previous studies have provided evidence of low-grade systemic inflammation in patients with metabolic syndrome (reviewed in [2]). In the present study, we carried out a detailed characterization of the different immune cell types and soluble inflammatory markers in metabolic syndrome and correlated some of these data with the circulating levels of glucose. The enhanced inflammatory status in metabolic syndrome that is reported herein has functional consequences, as illustrated for circulating platelet-bound leukocytes, which have increased adhesiveness to dysfunctional arterial endothelium, a prominent feature of the atherogenic process. The neutralization of the CX_3_CL1/CX_3_CR1 or CCL2/CCR2 axes partially diminished the initial adhesion of platelet-leukocyte and leukocyte adhesion to stimulated arterial endothelium, constituting a potential preventive target for cardiovascular events. 

Platelet activation is known to be associated with atherogenesis and cardiovascular morbidity [35]. Indeed, platelets express specific cell adhesion molecules upon their activation, such as P-selectin, and they release several inflammatory chemokines, including PF-4/CXCL4 or RANTES/CCL5 [35]. Platelet activation has previously been reported in metabolic syndrome [12,16,36,37], and we here confirm and extend these observations by showing positive correlations between the platelet activation state and blood glucose levels. Platelet surface molecules, such as GPIIb/IIIa (recognized by PAC-1) or P-selectin, are critically involved in the interaction of platelets with endothelial cells and leukocytes [35], all of which are central for atherosclerotic lesion formation. However, in contrast to previous reports [13,19], the circulating levels of sP-selectin, PF-4/CXCL4, or RANTES/CCL5 did not differ between the groups that were investigated here, which suggests a moderate thrombogenic profile in the metabolic syndrome patients.

We examined different leukocyte subtypes to gain insight into the immune state of the metabolic syndrome environment. Although no differences in the percentage of circulating neutrophils or platelet-neutrophil aggregates were detected between the patients and controls, a clear increase in the percentage of activated cells (CD11b expression) was observed in patient samples, which correlated with circulating glucose levels and suggests the potential existence of a proatherogenic state. This is consistent with our finding of increased circulating levels of IL-8, which is involved in neutrophil activation. By contrast, an increase in the number of circulating eosinophils was detected in metabolic syndrome patients, which correlated with the blood glucose levels. This finding of increased circulating eosinophils has been previously described and associated with impaired lung function in patients with metabolic syndrome [10]. These events were accompanied by elevations in platelet-eosinophil aggregates and eosinophil activation (CD11b expression). However, despite these findings, and, although eotaxins are known key eosinophil chemoattractants, we found no differences in the circulating levels of eotaxin-1/CCL11 and eotaxin-3/CCL26 between the groups, but circulating levels of eotaxin-2/CCL24 were significantly lower in the metabolic syndrome group. It is possible that other chemokines or other nonchemokine factors, such as complement factor C5a or platelet activating factor, might be responsible for the activation of this leukocyte subtype [38]. These intriguing observations may imply a yet unknown role for these cells in metabolic syndrome and warrant further investigation.

Human monocytes are known to be a heterogeneous cell population that is commonly classified into three subtypes: classical CD14^+^CD16^−^CCR2^+^ (Mon1), intermediate CD14^+^CD16^+^CCR2^+^ (Mon2), and nonclassical CD14^+^CD16^+^CCR2^−^ (Mon3) [39]. A previous study found no significant differences in the total number of circulating monocytes between the metabolic syndrome patients and healthy controls [17], which contrasts with our findings and those of another study [18]. Moreover, the increased percentage of total monocytes in patients with metabolic syndrome that is reported here positively correlated with circulating glucose levels. Closer examination revealed that this was due to the increase in the percentage of Mon2 and Mon3 monocytes as the percentage of Mon1 monocytes was diminished, as previously described [20]. In this context, increased numbers of Mon2 and Mon3 subtypes in hyperlipidemia has been associated with atherosclerosis development [40], and other studies have noted an enhancement in circulating CD16^+^ monocytes (Mon2 and Mon3) in CVD [41], which is possibly linked to disease outcome [42]. Furthermore, there is evidence to support that mobilized classical monocytes from the bone marrow mature into nonclassical monocytes through an intermediate subset. Along this line, an analysis of our results suggests that the increase of the Mon2 and Mon3 populations that are found in metabolic syndrome is likely due to a phenotypic shift of Mon1 monocytes (Figures S5 and S6, Supplemental data). However, how these different monocyte subtypes correlate with disease pathogenesis and clinical outcomes in metabolic syndrome is unknown. Of note, all of the monocyte subsets were found to be in an activated state (increased CD11b expression), and again this effect positively correlated with plasma glucose concentrations. Overall, our results suggest that activated monocytes in metabolic syndrome are more prone to interact with the dysfunctional endothelium and to release monocyte-derived inflammatory mediators, with the potential to initiate and amplify the atherogenic process.

The lymphoid lineage has been scarcely investigated in a metabolic syndrome environment. We show that, although an increased percentage of circulating CD3^+^ lymphocytes was detected in metabolic syndrome patients, as previously reported [18], both CD4^+^ and CD8^+^ cells were responsible for this enhancement. Additionally, a higher percentage of CD3^+^CD4^+^ and CD3^+^CD8^+^ lymphocyte-platelet aggregates was found in the metabolic syndrome patients than in controls. By contrast, the enhanced activation state of CD3^+^ lymphocytes in metabolic syndrome patients was mainly due to CD3^+^CD8^+^ cells. We also found that the percentages of circulating CD8^+^ cells, CD8^+^ cell-platelet aggregates, and CD8^+^CD69^+^ cells positively correlated with the blood glucose levels. In this context, the CD8^+^ cells are pro-atherogenic [43] and the CD8^+^ T-cell numbers in blood have been shown to correlate with the incidence of coronary events [44]. Thus, it is possible that CD8^+^ cells have a prominent role in the inflammatory status that is associated with metabolic syndrome. Indeed, some CD8^+^ cell-derived cytokines, such as TNFα, are significantly elevated in a metabolic syndrome scenario, whereas those that are derived from CD4^+^ lymphocytes either remained unchanged or decreased (IL-4) [45,46]. In regard to the latter observation, basophils and natural killer T cells are alternative sources of IL-4 [47,48], although an analysis of their circulating levels in this pathology has not been reported.

CX_3_CL1 receptor (CX_3_CR1) up-regulation is known to be associated with coronary artery disease [33] and with the development of CVD [24,49]. In this regard, two findings are worthy of mention. First, enhanced CX_3_CR1 expression was found in the platelets, platelet-neutrophil, -eosinophil, -Mon1 monocytes, and -CD8^+^ T cell aggregates, as well as in platelet-unbound Mon1 monocytes and CD8^+^ T lymphocytes of metabolic syndrome subjects. Second, positive correlations with circulating glucose concentrations were found for CX_3_CR1-expressing platelets, platelet-eosinophil, platelet-CD3^+^ T cell aggregates, and CD3^+^ T lymphocytes unbound to platelets. 

Activated platelets can mediate the endothelial adhesion of circulating leukocytes, a characteristic feature of dysfunctional endothelium [11]. Indeed, the augmented numbers of CX_3_CR1^+^ platelets are likely to be involved in the increased platelet-neutrophil, -eosinophil, -Mon1 monocytes, and -CD8^+^ T lymphocyte adhesion to the dysfunctional arterial endothelium in metabolic syndrome patients, but not in controls. In this line, significantly diminished leukocyte arrest was found in the absence of platelets, and the neutralization of CX_3_CL1 activity significantly impaired both platelet-leukocyte and leukocyte arterial arrest induced by TNFα to a similar extent. The analysis of these adhesive interactions suggests that CX_3_CL1 and CCL2 activity neutralization affect the endothelial arrest of the platelet-Mon1 monocyte aggregates and Mon1 monocytes, since these cells express both CX_3_CR1 and CCR2 receptors and the reductions in leukocyte adhesion are of a similar magnitude. Given this, it is tempting to speculate that the reduced percentage of circulating pro-inflammatory (Mon1) monocytes detected in metabolic syndrome patients would be the consequence of their adhesion to, and migration through, the dysfunctional arterial endothelium. Accordingly, therapeutic intervention of these chemokine/chemokine receptor axes may only be effective under pathological conditions. Despite these findings, soluble fractalkine/CX_3_CL1 circulating levels did not differ between the patients and controls, which is in agreement with a previous report [50]. 

In conclusion, we report that the low-grade systemic inflammation that is associated with metabolic syndrome is accompanied by a mild pro-thrombotic state with heightened platelet activation, which, together with the activation of different leukocyte subsets, results in the formation of platelet-leukocyte aggregates and their adhesion to dysfunctional arterial endothelium. The neutralization of CX_3_CL1/CX_3_CR1 or CCL2/CCR2 chemokine axes partly inhibits leukocyte adhesion through impaired proinflammatory monocyte (Mon1) adhesiveness to the dysfunctional endothelium, which suggests a potential link between the systemic inflammatory response and CVD development in metabolic syndrome. Finally, the positive correlations between glucose circulating levels and different circulating inflammatory mediators (TNFα) and the negative correlations with the anti-inflammatory cytokine IL-4 might be used as potential markers of CVD. Overall, it can be seen from metabolic syndrome that the modulation of the cellular inflammatory components in metabolic syndrome, especially Mon1 monocytes, might be crucial to prevent further cardiovascular complications.

## Figures and Tables

**Table 1:** Demographic and clinical features of metabolic syndrome patients and age-matched controls.

	Control Volunteers (*n* = 21)	Metabolic Syndrome Patients (*n* = 18)	*p* Value
Age (years)	48.8 ± 2.7	52.2 ± 3.2	0.42
Gender M/F (%)	5/16 (23.8/76.2)	3/15 (16.7/83.3)	0.59
BMI (kg/m^2^)	25.5 ± 0.7	31.0 ± 0.9 **	<0.01
Weight (kg)	69.4 ± 2.6	83.3 ± 2.9 **	<0.01
Height (cm)	164.9 ± 2.4	163.8 ± 1.7	0.71
Waist circumference (cm) total	85.4 ± 2.0	100.0 ± 1.7 **	<0.01
Waist circumference (cm) male	92.4 ± 2.1	105.0 ± 1.2 **	<0.01
Waist circumference (cm) female	83.0 ± 2.3	99.0 ± 1.9 **	<0.01
Systolic blood pressure (mmHg)	116.6 ± 2.0	130.3 ± 2.1 **	<0.01
Diastolic blood pressure (mmHg)	71.7 ± 1.9	79.9 ± 1.2 **	<0.01
Glucose (mg/dL)	86.7 ± 1.5	103.8 ± 3.0 **	<0.01
Insulin (mIU/L)	7.6 ± 0.9	18.4 ± 2.0 **	<0.01
HOMA Index	1.7 ±0.2	4.7 ± 0.5 **	<0.01
Total Cholesterol levels (mg/dL)	206.1 ± 6.8	239.5 ± 12.8 *	<0.05
LDL levels (mg/dL)	130.6 ± 5.4	156.2 ± 11.2 *	<0.05
HDL levels (mg/dL)	66.0 ± 2.5	50.5 ± 2.1 **	<0.01
Triglycerides (mg/dL)	81.0 ± 7.3	181.6 ± 30.0 **	<0.01
IgG (mg/dL)	966.7 ± 41.1	985.8 ± 48.9	0.76
IgM (mg/dL)	100.4 ± 7.6	104.5 ± 13.0	0.78
IgE (mg/dL)	42.6 ± 12.0	57.2 ± 17.3	0.48
CRP (mg/L)	1.4 ± 0.2	2.1 ± 0.4	0.23

BMI, body mass index; HOMA, homeostatic model assessment; LDL, low-density lipoprotein; HDL, high-density lipoprotein; Ig, immunoglobulin; CRP, C-reactive protein. Data are presented as mean ± SEM. * *p* < 0.05 or ** *p* < 0.01 relative to values in the control group.

## Supplementary Materials

The following are available online at https://www.mdpi.com/2077-0383/8/5/708/s1, Figure S1: Gating strategy for human platelets in whole blood according to morphological properties and CD41 detection by flow cytometry, Figure S2: Gating strategy for human neutrophils and eosinophils in whole blood according to morphological properties and CD16 expression by flow cytometry, Figure S3: Gating strategy for human monocyte detection in whole blood by flow cytometry, Figure S4: Gating strategy for human T lymphocyte detection in whole blood by flow cytometry, Figure S5: Flow cytometry detection and morphologic gating of human monocytes for the immunophenotype study of the different monocyte subsets, Figure S6: Comparison of CD14, CD16, CCR2 and CX3CR1 expression in the different monocyte subsets of MS patients and healthy volunteers, Table S1: Differential markers of monocyte subpopulations, Table S2: Number of patients that met each of the 5 metabolic syndrome criteria.
