# Supplementary material for: Systemic Inflammation in Metabolic Syndrome: Increased Platelet and Leukocyte Activation, and Key Role of CX3CL1/CX3CR1 and CCL2/CCR2 Axes in Arterial Platelet-Proinflammatory Monocyte Adhesion
Source: J Clin Med. 2019 May 18;8(5):708. doi: 10.3390/jcm8050708 (PMC6572270; doi:10.3390/jcm8050708)
Supplement: Supplementary file 1 [file jcm-08-00708-s001.pdf]

# Systemic Inflammation in Metabolic Syndrome: Increased Platelet and Leukocyte Activation, and Key Role of $CX_3CL1/CX_3CR1$ and CCL2/CCR2 Axes in Arterial Platelet-Proinflammatory Monocyte Adhesion.

[1,3]Patrice Marques[#], [1,3]Aida Collado[#], [2,3,4,5]Sergio Martinez-Hervás, [1,3]Elena Domingo, [3,4,5]Ester Benito, [1,3,5]Laura Piqueras, [2,3,4,5]José T. Real, [2,3,4,5]Juan F. Ascaso*, [1,3,5]Maria-Jesus Sanz*

[1]Department of Pharmacology and [2]Department of Medicine, Faculty of Medicine and Odontology, University of Valencia, Av. Blasco Ibáñez 15, 46010, Valencia, Spain. [3]Institute of Health Research INCLIVA, [4]Endocrinology and Nutrition Service, University Clinic Hospital of Valencia, Av. Menéndez Pelayo 4, 46010, Valencia, Spain and [5]CIBERDEM-Spanish Biomedical Research Centre in Diabetes and Associated Metabolic Disorders, ISCIII, Av. Monforte de Lemos 3-5, 28029, Madrid, Spain.

## Materials and Methods

### *Cell Culture*

Human umbilical arterial endothelial cells (HUAEC) were isolated by collagenase treatment [1] and maintained in human endothelial cell specific medium (EBM-2, Lonza, Barcelona, Spain), supplemented with endothelial growth media (EGM-2, Lonza) and containing 10% fetal bovine serum (FBS; Biowest, Nuaillé, France). Cells were grown to confluence up to passage 1 to preserve endothelial features. Prior to every experiment, cells were incubated for 24 h in medium containing 2% FBS.

### *Flow Cytometry*

Blood samples for flow cytometry studies were collected in BD Vacutainer® blood collection tubes containing 3.2% sodium citrate, or in BD Vacutainer® PST™ II tubes with lithium/heparin (17 IU/mL) as anticoagulant agents (both from BD Biosciences, San Jose, CA). All samples were run in a FACSVerse™ flow cytometer (BD Biosciences) and all flow cytometry data were analyzed with FlowJo® v10.0.7 software (FlowJo LLC, Ashland, OR).

To determine platelet activation, PAC-1$^+$ platelets (detecting activated integrin $\alpha_{IIb}\beta_3$/GPIIb/IIIa) and the expression of P-selectin (CD62P) were measured in platelets by flow cytometry. Citrated blood samples (6.25 µL) were diluted 1:10 in glucose buffer (1 mg/mL glucose in phosphate buffered saline containing 0.35% bovine serum albumin; Sigma-Aldrich, Madrid, Spain). Samples were incubated in the dark for 30 min with a 5-carboxyfluorescein (CF)-Blue™-conjugated monoclonal antibody (mAb) against human CD41 (1.25 µL, clone HIP8, $IgG_1$; Immunostep, Salamanca, Spain) and a fluorescein isothiocyanate (FITC)-conjugated mouse mAb against the human integrin $\alpha_{IIb}\beta_3$/GPIIb/IIIa (2.5 µL, clone PAC-1, IgM; BD Biosciences), or with an allophycocyanin (APC)-conjugated mAb against human P-selectin (1.25 µL, clone HI62P, $IgG_1$; Immunostep). The CD41$^+$ population (platelets) was selected according to the gating strategy illustrated in Figure S1 and expressed as the percentage of positive platelets.

To determine the grade of leukocyte activation, the expression of CD11b was analyzed on circulating neutrophils, eosinophils and monocyte subsets, or CD69 expression on $CD3^+$ T cells and lymphocyte subsets. Heparinized whole blood samples were incubated in the dark for 30 min with saturated amounts of a phycoerythrin (PE)-conjugated mAb against human integrin CD11b (clone CBRM1/5, $IgG_1$; Biolegend, San Diego, CA) or a PE-conjugated mAb against human CD69 (clone FN50, $IgG_1$; Immunostep). Fractalkine/$CX_3CL1$ receptor ($CX_3CR1$) expression was determined using a PE-conjugated rat mAb against human $CX_3CR1$ (clone 2A9-1, $IgG_{2B}$; Biolegend). In some experiments, heparinized blood samples were incubated with ethylenediaminetetraacetic acid (EDTA, 10 mM, for 15 min at 37ºC) to promote platelet dissociation as described [2]. This disaggregation was measured using the marker CD41 in circulating leukocyte subsets. To do this, heparinized whole blood or EDTA samples were incubated in the dark for 30 min with saturated amounts of a PE/Cy™7-conjugated mouse mAb against human CD41 (clone HIP8, $IgG_1$; Biolegend) or a CF-Blue™-conjugated mAb against human CD41 (clone HIP8, $IgG_1$; Immunostep).

Red blood cells were lysed using a commercial lysis buffer (BD FACS™ lysing solution 10× concentrate; BD Biosciences).

The expression of the different markers was determined on $CD16^+$ (neutrophils, Figure S2), $CD16^-$ (eosinophils, Figure S2), $CD14^+$ (monocytes, Table S2, Figure S3), $CD3^+$ (T lymphocytes, Figure S4), $CD8^+$ (cytotoxic lymphocytes, Figure S4), and $CD4^+$ (T helper lymphocytes Figure S4). Gating strategies are illustrated in Figures S2–S4.

***Quantification of Soluble Inflammatory and Metabolic Markers***

Heparinized human whole blood (17 IU heparin/mL) was collected and centrifuged to obtain the plasma, which was stored at -80ºC. The following cytokines, chemokines and adipokines were measured in plasma samples by enzyme-linked immunosorbent assay (ELISA; DuoSet® ELISA Development Systems, R&D Systems, Abingdon, UK) as previously described [3, 4]: human soluble interleukin (IL)-4, IL-6, IL-10, IL-12, IL-13, IL-25, IL-33, tumor necrosis factor

(TNF)-$\alpha$, interferon (IFN)-$\gamma$, growth-regulated oncogene-$\alpha$ (GRO$\alpha$/CXCL1), platelet factor-4 (PF-4/CXCL4), IL-8/CXCL8, monocyte chemoattractant protein-1 (MCP-1/CCL2), regulated on activation normal T cell expressed and secreted chemokine (RANTES/CCL5), eotaxin-1/CCL11, eotaxin-2/CCL24, eotaxin-3/CCL26, fractalkine/$CX_3CL1$ and soluble P-selectin (sP-selectin). Results were expressed as pg or ng/mL of mediator in plasma.

***Leukocyte-Endothelial Cell interactions Under Flow Conditions***

Before starting each assay, whole blood was diluted 1:10 with Hank's balanced salt solution (HBSS; Lonza, Barcelona, Spain) without calcium or magnesium at 37ºC. Blood was perfused across HUAEC monolayers, unstimulated or stimulated with 20 ng/mL TNF$\alpha$ (Sigma-Aldrich, Madrid, Spain) for 24 h. Some cells were incubated with a monoclonal neutralizing antibody against human $CX_3CL1$ (5 μg/mL, $IgG_1$; ref: MAB3652, R&D Systems, Abingdon, UK), neutralizing antibody against human CCL2 (2 μg/mL, $IgG_1$; ref: MAB279, R&D Systems) or with an isotype-matched control antibody (MOPC-21, 2 μg/mL, $IgG_1$; ref: M5284, Sigma-Aldrich) 10 min before blood perfusion. Again, experiments were performed in heparinized blood treated or not with EDTA (10 mM, for 15 min, 37ºC) to promote platelet dissociation.

In all experiments, leukocyte interactions were determined after 7 min at 0.5 dyn/$cm^2$. Cells interacting on the surface of the endothelium were visualized and recorded (×20 objective, ×10 eyepiece) using a phase-contrast microscopy (Axio Observer A1 Carl Zeiss microscope; Carl Zeiss, Thornwood, NY). For each determination, at least 5 fields were recorded for 10 s and then averaged. Finally, recorded images were saved on a computer for further analysis.

***Immunofluorescence studies***

To visualize adherent $CX_3CR1$ and CCR2 expressing-platelet–leukocyte complexes, platelet–monocyte aggregates, leukocytes or monocytes with endothelial cells we performed an immunofluorescence analysis. Confluent endothelial cells were grown on glass coverslips and stimulated with 20 ng/mL TNF$\alpha$, for 24 h. Heparinized blood from patients with metabolic syndrome and age-matched controls was incubated without or with EDTA. After the flow

chamber assay, cells were fixed with 4% paraformaldehyde and blocked in PBS containing 1% BSA (Sigma-Aldrich, Madrid, Spain). Subsequently, cells were incubated at room temperature for 2 h with an Alexa Fluor 594-conjugated antibody against human CD45, to detect leukocytes (1:50 dilution, red, clone RA3-6B2, $IgG_{2A}$, Biolegend, San Diego, CA) or an APC-conjugated antibody against human CD14, to detect monocytes (1:50 dilution, red, clone 47-3D6, $IgG_{2A}$, Immunostep, Salamanca, Spain). Additionally, all cells were also incubated for 2 h with an Alexa Fluor 350-conjugated antibody against human CCR2 (1:50 dilution, blue, clone 48607, $IgG_{2b}$, R&D Systems, Abingdon, UK) and a FITC-conjugated antibody against human $CX_3CR1$ (1:50 dilution, green, clone 528728, $IgG_1$, R&D Systems). All antibody dilutions were made in 0.1% BSA/PBS. Images were captured with Zeis Axio Observer A1 fluorescence microscope (Zeiss, Thornwood, NY).

## References


1. Rius C., Piqueras, L., Gonzalez-Navarro, H., Albertos, F., Company, C., Lopez-Gines, C., Ludwig, A., Blanes, J.I., Morcillo, E.J., and Sanz, M.J. Arterial and venous endothelia display differential functional fractalkine (CX3CL1) expression by angiotensin-II. *Arterioscler Thromb Vasc Biol.* **2013**, 33,96-104, DOI: 10.1161/ATVBAHA.112.254870.,
2. Postea O., Vasina, E.M., Cauwenberghs, S., Projahn, D., Liehn, E.A., Lievens, D., Theelen, W., Kramp, B.K., Butoi, E.D., Soehnlein, O., Heemskerk, J.W., Ludwig, A., Weber, C., and Koenen, R.R. Contribution of platelet CX(3)CR1 to platelet-monocyte complex formation and vascular recruitment during hyperlipidemia. *Arterioscler Thromb Vasc Biol.* **2012**, 32,1186-1193, DOI: 10.1161/ATVBAHA.111.243485.
3. Furio E., Garcia-Fuster, M.J., Redon, J., Marques, P., Ortega, R., Sanz, M.J., and Piqueras, L. CX3CR1/CX3CL1 Axis Mediates Platelet-Leukocyte Adhesion to Arterial Endothelium in Younger Patients with a History of Idiopathic Deep Vein Thrombosis. *Thromb Haemost.* **2018**, 118,562-571, DOI: 10.1055/s-0038-1629897.
4. Marques P., Collado, A., Escudero, P., Rius, C., Gonzalez, C., Servera, E., Piqueras, L., and Sanz, M.J. Cigarette Smoke Increases Endothelial CXCL16-Leukocyte CXCR6 Adhesion In Vitro and In Vivo. Potential Consequences in Chronic Obstructive Pulmonary Disease. *Front Immunol.* **2017**, 8,1766, DOI: 10.3389/fimmu.2017.01766.

**Table S1.** Differential markers of monocyte subpopulations.

| Marker | Cellular Population |
|---|---|
| $CD14^+CD16^-CCR2^+$ | Monocyte type 1 (Mon1) |
| $CD14^+CD16^+CCR2^+$ | Monocyte type 2 (Mon2) |
| $CD14^+CD16^+CCR2^-$ | Monocyte type 3 (Mon3) |

**Table S2.** Number of patients that met each of the 5 metabolic syndrome criteria. Ig, immunoglobulin; CRP, C-reactive protein. Data are presented as mean ± SEM. *$p < 0.05$ relative to values in the control group.

| | | Number of Patients (%) | Non-Metabolic Parameters | Age-Matched Controls | Metabolic Syndrome Patients | *p* Value |
|---|---|---|---|---|---|---|
| Criteria | **Abdominal obesity** | 18/18 (100%) | Age (years) | 48.8 ± 2.7 | 52.2 ± 3.2 | 0.42 |
| | | | Gender M/F (%) | 5/16 (23.8/76.2) | 3/15 (16.7/83.3) | 0.59 |
| | | | IgG (mg/dL) | 966.7 ± 41.1 | 985.8 ± 48.9 | 0.76 |
| | | | IgM (mg/dL) | 100.4 ± 7.6 | 104.5 ± 13.0 | 0.78 |
| | | | IgE (mg/dL) | 42.6 ± 12.0 | 57.2 ± 17.3 | 0.48 |
| | | | CRP (mg/L) | 1.4 ± 0.2 | 2.1 ± 0.4 | 0.23 |
| | **Arterial hypertension** | 11/18 (61.1%) | Age (years) | 48.8 ± 2.7 | 53,2 ± 3.9 | 0.35 |
| | | | Gender M/F (%) | 5/16 (23.8/76.2) | 1/10 (9.1/90.9) | 0.39 |
| | | | IgG (mg/dL) | 966.7 ± 41.1 | 1101.0 ± 45.7 | 0.05 |
| | | | IgM (mg/dL) | 100.4 ± 7.6 | 121.9 ± 17.3 | 0.20 |
| | | | IgE (mg/dL) | 42.6 ± 12.0 | 52.1 ± 22.4 | 0.47 |
| | | | CRP (mg/L) | 1.4 ± 0.2 | 2.2 ± 0.4 | 0.17 |
| | **Dysglycemia** | 12/18 (66.7%) | Age (years) | 48.8 ± 2.7 | 55.9 ± 3.5 | 0.12 |
| | | | Gender M/F (%) | 5/16 (23.8/76.2) | 2/10 (16.7/83.3) | 0.64 |
| | | | IgG (mg/dL) | 966.7 ± 41.1 | 1021.0 ± 60.5 | 0.45 |
| | | | IgM (mg/dL) | 100.4 ± 7.6 | 104.7 ± 18.5 | 0.80 |
| | | | IgE (mg/dL) | 42.6 ± 12.0 | 37.3 ± 8.1 | 0.58 |
| | | | CRP (mg/L) | 1.4 ± 0.2 | 1.6 ± 2.7 | 0.87 |
| | **Hypertriglycleridemia** | 12/18 (66.7%) | Age (years) | 48.8 ± 2.7 | 51.3 ± 3.8 | 0.58 |
| | | | Gender M/F (%) | 5/16 (23.8/76.2) | 3/9 (25.0/75.0) | > 0.99 |
| | | | IgG (mg/dL) | 966.7 ± 41.1 | 957.9 ± 51.6 | 0.90 |
| | | | IgM (mg/dL) | 100.4 ± 7.6 | 92.9 ± 10.7 | 0.56 |
| | | | IgE (mg/dL) | 42.6 ± 12.0 | 62.3 ± 24.4 | 0.90 |
| | | | CRP (mg/L) | 1.4 ± 0.2 | 2.5 ± 0.6 | 0.13 |
| | **Low HDL levels** | 9/18 (50.0%) | Age (years) | 48.8 ± 2.7 | 49.4 ± 5.3 | 0.90 |
| | | | Gender M/F (%) | 5/16 (23.8/76.2) | 2/7 (22.2/77.8) | > 0.99 |
| | | | IgG (mg/dL) | 966.7 ± 41.1 | 934.0 ± 84.3 | 0.70 |
| | | | IgM (mg/dL) | 100.4 ± 7.6 | 128.3 ± 23.0 | 0.14 |
| | | | IgE (mg/dL) | 42.6 ± 12.0 | 53.4 ± 24.6 | 0.81 |
| | | | CRP (mg/L) | 1.4 ± 0.2 | 3.1 ± 0.7* | < 0.05 |

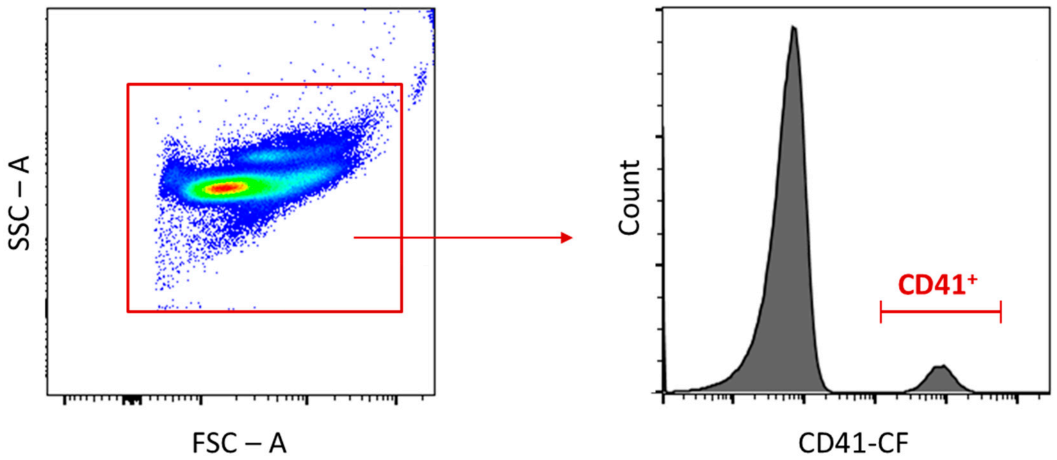


**Figure S1.** Gating strategy for human platelets in whole blood according to morphological properties and CD41 detection by flow cytometry. Platelets were gated according to a low side scatter (SSC-A) and forward scatter (FSC-A) in a logarithmic scale and defined as $CD41^+$ population.

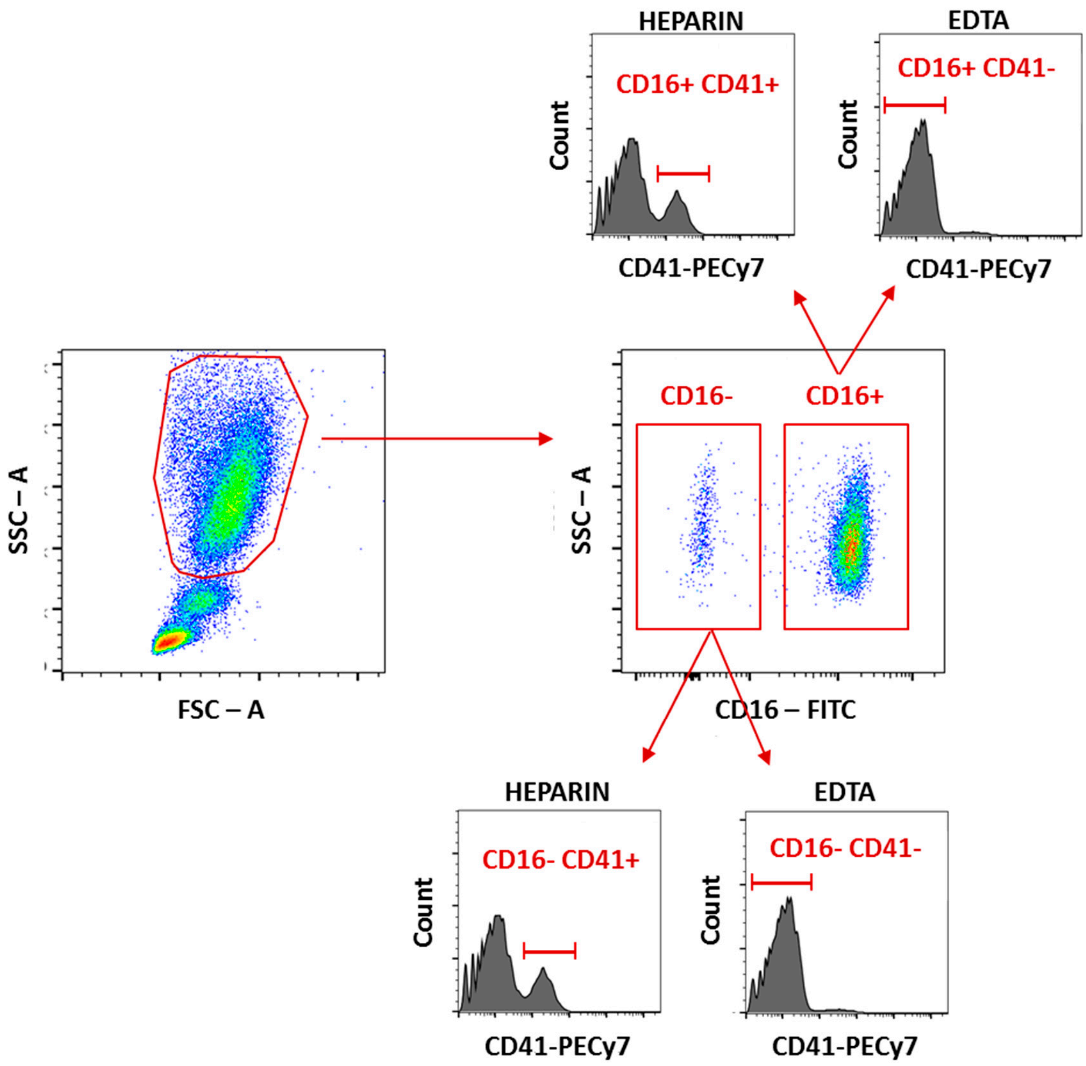


**Figure S2.** Gating strategy for human neutrophils and eosinophils in whole blood according to morphological properties and CD16 expression by flow cytometry. Populations were selected by morphology (high SSC-A). A CD16 antibody was used to detect neutrophils (CD16$^+$) and eosinophils (CD16$^-$). In heparinized blood, neutrophil-platelet-aggregates were selected as a CD16$^+$CD41$^+$ population and eosinophil-platelet aggregates as a CD16$^-$CD41$^+$ population.

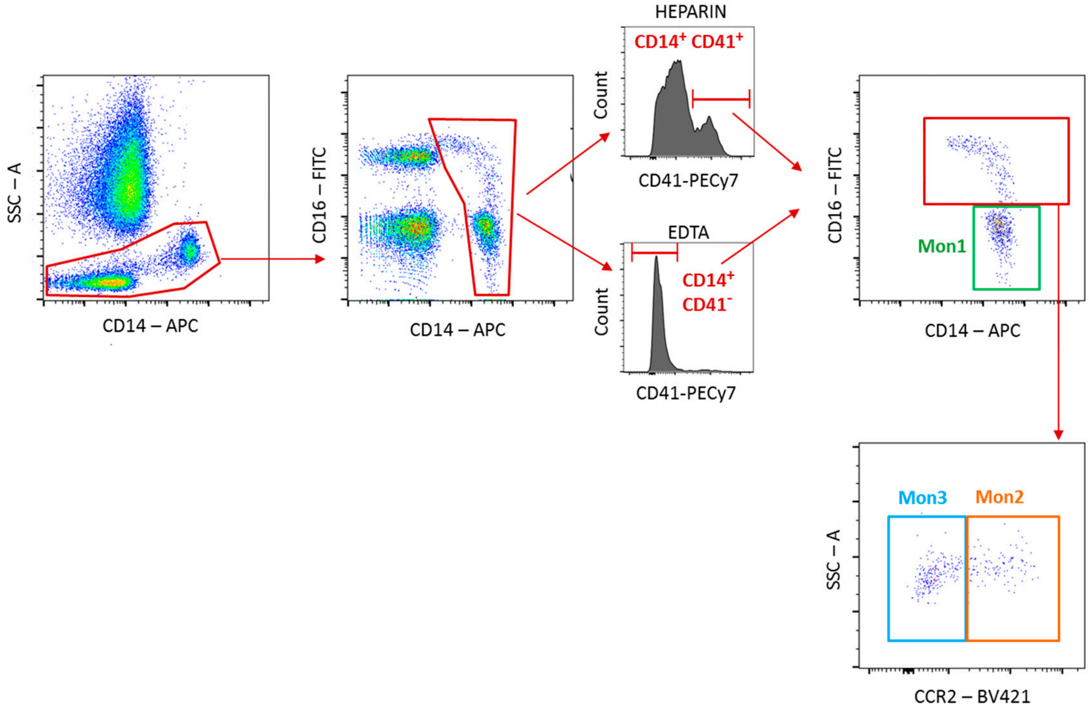


**Figure S3.** Gating strategy for human monocyte detection in whole blood by flow cytometry. Monocytes were selected by CD14 labelling and morphology (medium SSC-A). For the detection of monocyte subpopulations, CD16 and CCR2 markers were used. Monocytes-platelets complexes were selected as $CD14^{+}CD41^{+}$ populations in heparinized whole blood, and platelet-free monocytes were gated as $CD14^{+}CD41^{-}$ from blood incubated with EDTA.

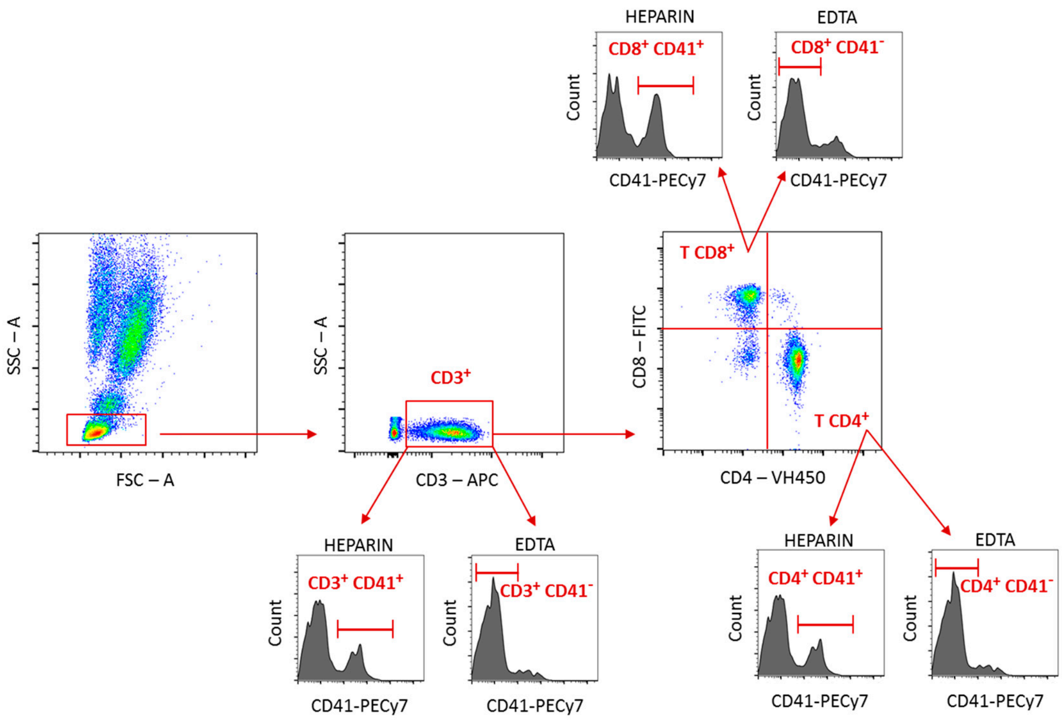


**Figure S4**. Gating strategy for human T lymphocyte detection in whole blood by flow cytometry**.** T lymphocytes were selected as a CD3$^{+}$ population and with a low SSC-A. T helper (Th) lymphocytes were selected as the CD3$^{+}$CD4$^{+}$ population. In heparinized blood, Th lymphocyte-platelet complexes were selected as the CD3$^{+}$CD4$^{+}$CD41$^{+}$ population, whereas platelet-free Th lymphocytes were gated as CD3$^{+}$CD4$^{+}$CD41$^{−}$ from blood incubated with EDTA. Cytotoxic lymphocytes were selected as CD3$^{+}$CD8$^{+}$. In heparinized blood, cytotoxic lymphocyte-platelet complexes were selected as the CD3$^{+}$CD8$^{+}$CD41$^{+}$ population, whereas platelet free cytotoxic lymphocytes were selected as CD3$^{+}$CD8$^{+}$CD41$^{-}$ from blood incubated with EDTA**.**

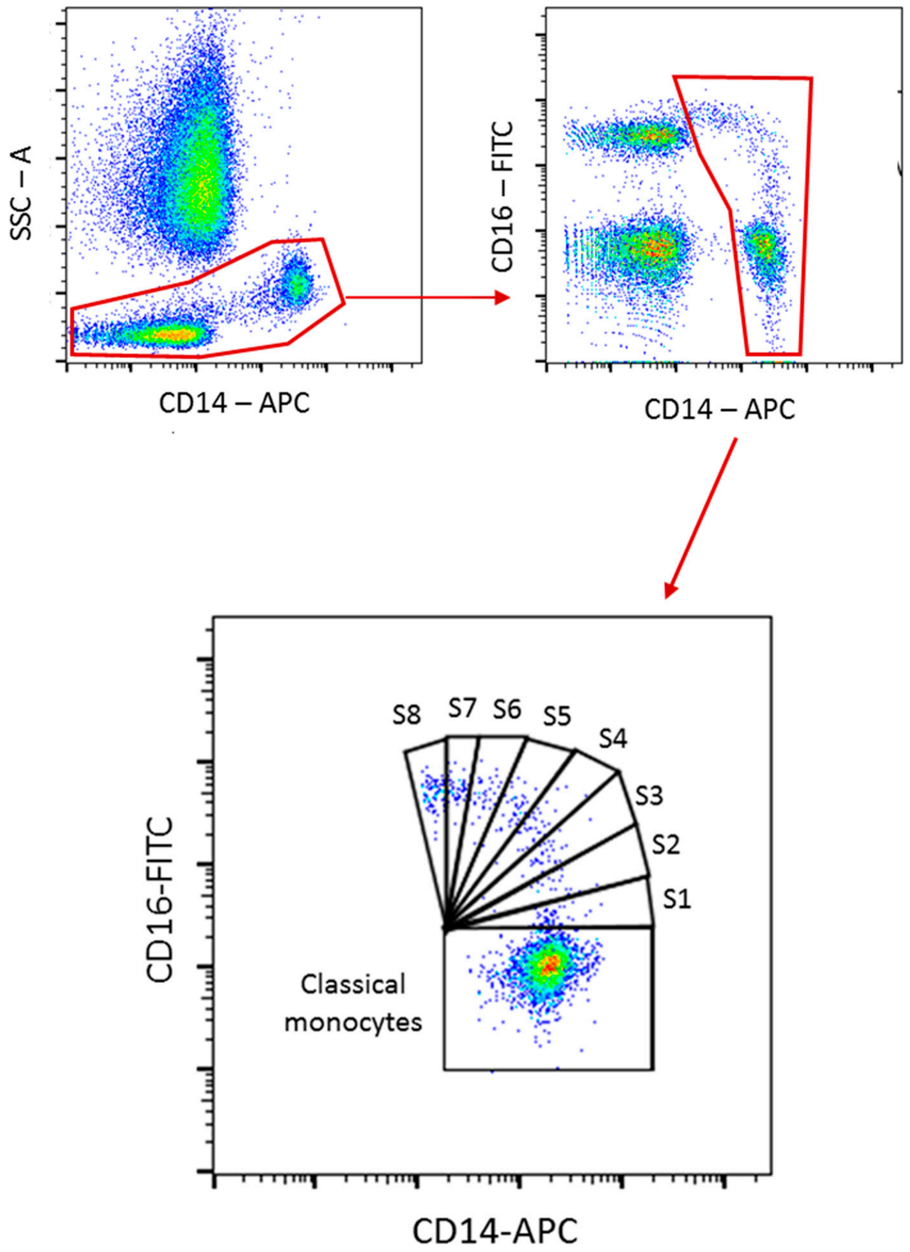


**Figure S5.** Flow cytometry detection and morphologic gating of human monocytes for the immunophenotype study of the different monocyte subsets. Classical monocytes (Mon1) were selected as $CD14^{+}CD16^{-}$ population from whole blood. In order to study the monocyte immunophenotypic changes, eight gates were designed (S1-8) from $CD14^{+}CD16^{-}$ to $CD14^{low}CD16^{+}$.

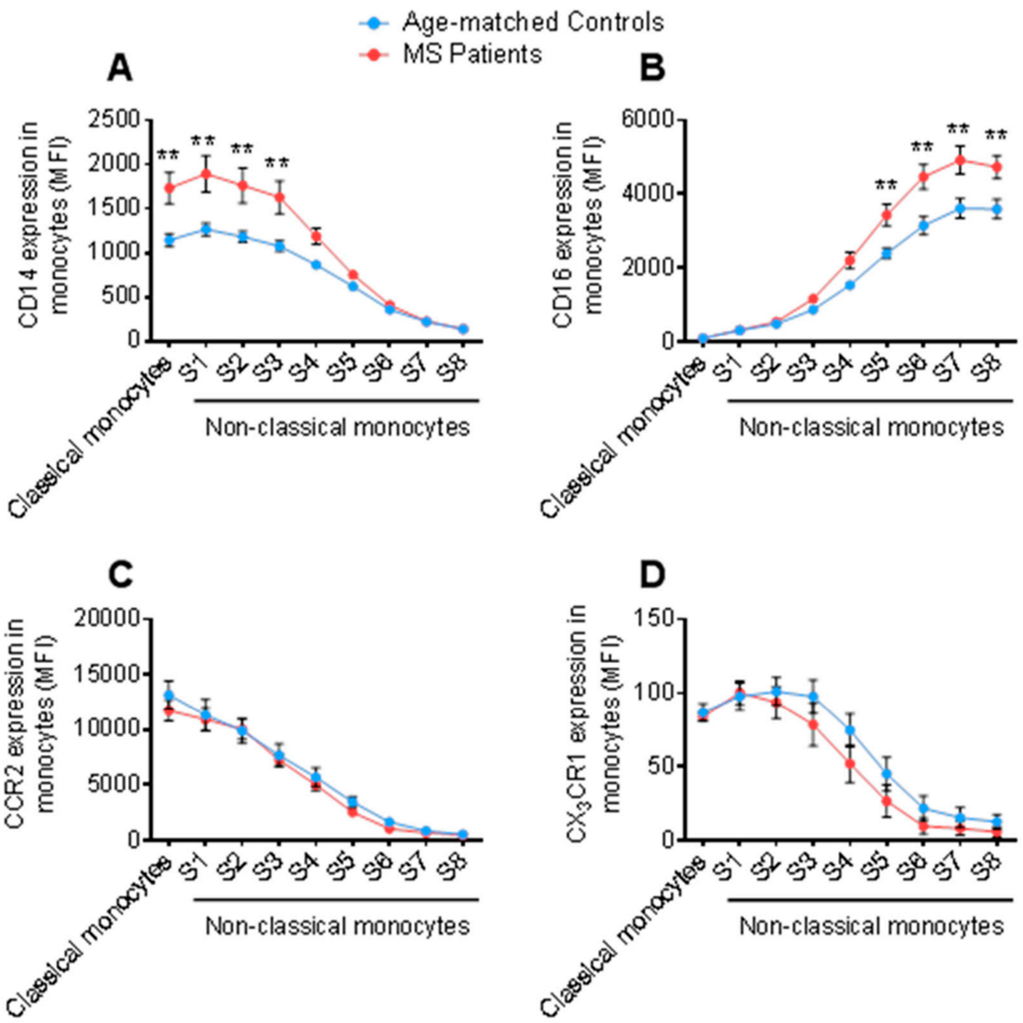


**Figure S6.** Comparison of CD14, CD16, CCR2 and $CX_3CR1$ expression in the different monocyte subsets of MS patients and healthy volunteers**.** Immunophenotypic changes of the different monocyte subsets, relative to CD14 (**A**), CD16 (**B**), CCR2 (**C**) and $CX_3CR1$ (**D**) expression in metabolic syndrome patients and age-matched controls. $^{**}p < 0.01$ relative to values in the control group.
